# Programmable System of Cas13-Mediated RNA Modification and Its Biological and Biomedical Applications

**DOI:** 10.3389/fcell.2021.677587

**Published:** 2021-07-27

**Authors:** Tian Tang, Yingli Han, Yuran Wang, He Huang, Pengxu Qian

**Affiliations:** ^1^Center of Stem Cell and Regenerative Medicine, and Bone Marrow Transplantation Center of the First Affiliated Hospital, Zhejiang University School of Medicine, Hangzhou, China; ^2^Institute of Hematology, Zhejiang University & Zhejiang Engineering Laboratory for Stem Cell and Immunotherapy, Hangzhou, China; ^3^Zhejiang Laboratory for Systems & Precision Medicine, Zhejiang University Medical Center, Hangzhou, China; ^4^Dr. Li Dak Sum & Yip Yio Chin Center for Stem Cell and Regenerative Medicine, Zhejiang University, Hangzhou, China

**Keywords:** CRISPR-Cas13 system, RNA modification, epigenetic editing, CRISPR, cell biology

## Abstract

Clustered regularly interspaced short palindromic repeats (CRISPR)-Cas13 has drawn broad interest to control gene expression and cell fate at the RNA level in general. Apart from RNA interference mediated by its endonuclease activity, the nuclease-deactivated form of Cas13 further provides a versatile RNA-guided RNA-targeting platform for manipulating kinds of RNA modifications post-transcriptionally. Chemical modifications modulate various aspects of RNA fate, including translation efficiency, alternative splicing, RNA–protein affinity, RNA–RNA interaction, RNA stability and RNA translocation, which ultimately orchestrate cellular biologic activities. This review summarizes the history of the CRISPR-Cas13 system, fundamental components of RNA modifications and the related physiological and pathological functions. We focus on the development of epi-transcriptional editing toolkits based on catalytically inactive Cas13, including RNA Editing for Programmable A to I Replacement (REPAIR) and xABE (adenosine base editor) for adenosine deamination, RNA Editing for Specific C-to-U Exchange (RESCUE) and xCBE (cytidine base editor) for cytidine deamination and dm^6^ACRISPR, as well as the targeted RNA methylation (TRM) and photoactivatable RNA m^6^A editing system using CRISPR-dCas13 (PAMEC) for m^6^A editing. We further highlight the emerging applications of these useful toolkits in cell biology, disease and imaging. Finally, we discuss the potential limitations, such as off-target editing, low editing efficiency and limitation for AAV delivery, and provide possible optimization strategies.

## Introduction

### Type VI (Cas13) Clustered Regularly Interspaced Short Palindromic Repeats (CRISPR)-Cas Systems

Prokaryotic clustered regularly interspaced short palindromic repeats (CRISPR) RNAs and CRISPR-associated (Cas) proteins perform adaptive immune functions that protect prokaryotic cells by degrading invading genetic material, such as bacteriophages and exogenous plasmids (Wiedenheft et al., 2009). Once the foreign DNA invades bacteria and archaea, the CRISPR adaptation machinery system recognizes and captures a small piece of the invader that is integrated into a CRISPR array. The transcription of the CRISPR array and processing then generate mature CRISPR RNAs (CrRNAs) relying on a series of Cas proteins and/or host factors. In addition to immune adaptation and CrRNA biogenesis, CrRNA and catalytic Cas proteins form an effector complex that cleaves and promotes the degradation of identified nucleic acid materials, which prevents further infection (Wiedenheft et al., 2012). In light of Cas protein composition, the CRISPR-Cas system is divided into the following two classes: Class 1 depends on multiple Cas effectors, whereas Class 2 utilizes a single effector protein. Based on the constitution and architecture of CRISPR-Cas locus, each class can be further classified into multiple types and subtypes (Makarova et al., 2015). Type II is one of the most pervasive types and is identified by the single-component effector protein Cas9, which is renowned for efficient and specific DNA interference (Platt et al., 2014; Shalem et al., 2014; Chen et al., 2015; Patel et al., 2017; Daniloski et al., 2021). Certain Cas9 orthologs can target RNA in addition to their ability to cleave DNA (Price et al., 2015; Nelles et al., 2016; Rousseau et al., 2018; Marina et al., 2020; Batra et al., 2021). Some of them mediate RNA manipulation independent of a protospacer-adjacent motif (PAM, 5′-NGG-3′) (Rousseau et al., 2018; Strutt et al., 2018; Marina et al., 2020).

Type VI CRISPR proteins containing naturally RNA-targeting endonucleases were recently discovered. Based on the phylogeny of Cas13 protein, the CRISPR-Cas13 system is classified into Cas13a (also known as C2C2), Cas13b, Cas13c, Cas13d, Cas13x, and Cas13y (Shmakov et al., 2015; Konermann et al., 2018; Yan et al., 2018; O’Connell, 2019; Xu et al., 2021; Table 1). All of them require two conserved Higher Eukaryotes and Prokaryotes Nucleotide-binding (HEPN) domains to mediate RNA-guided single-strand RNA degradation. *Leptotrichia shahii* C2c2 (LshC2C2) is the first identified single-component effector protein. Abudayyeh et al. (2016) showed that Protospacer flanking site sequence (PFS, a non-G motif immediately flanking the 3′ end of the protospacer), RNA secondary structure and the seed region of CrRNA sequence were critical for nuclease activity of LshC2C2 in bacteria. However, Cas13a from *Leptotrichia wadei* (LwaCas13a) does not rely on the PFS motif and exhibits more robust targeting efficiency than *Leptotrichia shahii* Cas13a (LshCas13a). They both perform a “collateral effect” of promiscuous RNase activity in bacteria upon CrRNA base-pairing with targeted transcripts. Due to this feature, LwaCas13a and other orthologs of LshCas13a have been elegantly programmed to platforms termed Specific High Sensitivity Enzymatic Reporter UnLOCKing (SHERLOCK) and SHERLOCKv2, which have been used to detect multiple strains of Zika and Dengue virus, identify bacterial pathogens, genotype human DNA, and detect mutations in cell-free DNA from cancer patients (Gootenberg et al., 2018; Kellner et al., 2019; Shen et al., 2020). To assess the knockdown efficiency of fluorescence without the msfGFP component, 21 orthologs of Cas13a, 15 of Cas13b, and seven of Cas13c were adapted for expression in mammalian cells, from which Cas13b from *Prevotella* sp. *P5-125* (PspCas13b) stood out because of its highest efficiency and accuracy. PspCas13b monitors RNA cleavage without PFS constraints but with high fidelity in mammalian cells, which can be used for further applications (Abudayyeh et al., 2017). Cas13d from *Ruminococcus flavefaciens* strain XPD3002 (CasRx) is small enough to be packaged in AAV delivery system with a median size of 930 amino acids and has one of the most robust endogenous knockdown efficiencies without PFS limitation and detectable off-target performance (CasRx average >90%, LwaCas13a-average >60%) (Abudayyeh et al., 2017). Recently, two compact families of CRISPR-Cas ribonucleases, named Cas13x and Cas13y (775 to 803 amino acids), were identified. Cas13x.1 is the smallest effector to date, which is nearly 200 amino acids smaller than CasRx. Moreover, Cas13x.1 shows comparable efficiency and specificity as CasRx according to RNA knock-down assays on a large number of endogenous genes (Xu et al., 2021). Therefore, these characteristics allow Cas13x or dCas13x to be ideal modifiable RNA-targeting platforms for further transcriptome engineering.

**TABLE 1:** Major subtypes of Type-VI CRISPR/Cas system and their characteristics.

**Subtype**	**Represent**	**Size**	**Specificity**	**Efficiency**	**Application**
Cas13a	LwaCas13a	1180 aa	High	57.5% for KRAS	RNA knockdown, imaging, nucleic acid detection
Cas13b	PspCas13b	1127 aa	High	62.9% for KRAS	RNA knockdown, imaging, epigenetic engineering
Cas13c	FpCas13c	1121 aa	Unclear	Low	Unclear
Cas13d	CasRx	967 aa	High	>90% for KRAS	RNA knockdown, imagine, Epigenetic engineering, circRNA screening
Cas13x	Cas13x.1	775 aa	High	>90% for KRAS	RNA knockdown, epigenetic engineering
Cas13y	Cas13y.1	790 aa	High	>90% for KRAS	RNA knockdown

One of the major concerns and ramifications of gene therapy applications based on CRISPR-Cas13 is that Cas13 does not cause permanent variation in the genetic code in contrast to CRISPR-Cas9. Therefore, consistently importing exogenous effectors are required to achieve or maintain therapeutic efficacy, which may trigger a severe immune response. However, they still exhibit several strengths. Firstly, as RNA manipulation is tunable in yield and reversible in time, Cas13 platforms might be substitutional options for DNA-correction strategies, which cause severe or even lethal side-effects (Vogel and Stafforst, 2019). For example, when microsatellite repeat expansions were targeted by genome editing, DNA breaks near to the repeats activated the repair machinery of which the activity was linked to expansion growth and might cause further mutation of the repeats. In addition, the transient nature of RNA engineering approaches will likely be feasible for treating diseases caused by temporary changes in cell state, such as local inflammation, and disorders through modifying the function of proteins involved in disease-related signal transduction (Cox et al., 2017). Secondly, RNA-targeting therapeutic interventions are more symptom-targeted for diseases caused by RNA-level abnormalities. Many genetic diseases, such as cystic fibrosis, frontotemporal dementia and tumor progression, originate from splicing variation which leads to gain-of-function mutations or loss of function mutations (Garcia-Blanco et al., 2004; Wang et al., 2021). The accumulation of the repeat containing transcripts into abnormal RNA foci in the nucleus through multivalent base-pairing is a pathogenic feature of several neurological and neuromuscular disorders, including Huntington’s disease and Myotonic dystrophy type I (DM1) (Jain and Vale, 2017; Batra et al., 2021). So changing the splicing forms, disrupting RNA–RNA base pairing or eliminating these toxic RNA by RNA editing or degrading platform are possible strategies for treating relative disease. Thus, the Cas13 system holds great potentials for gene therapy, but needs to be carefully designed.

### RNA Modifications

#### *N*^6^-Methyladenosine (m^6^A)

**FIGURE 1:**
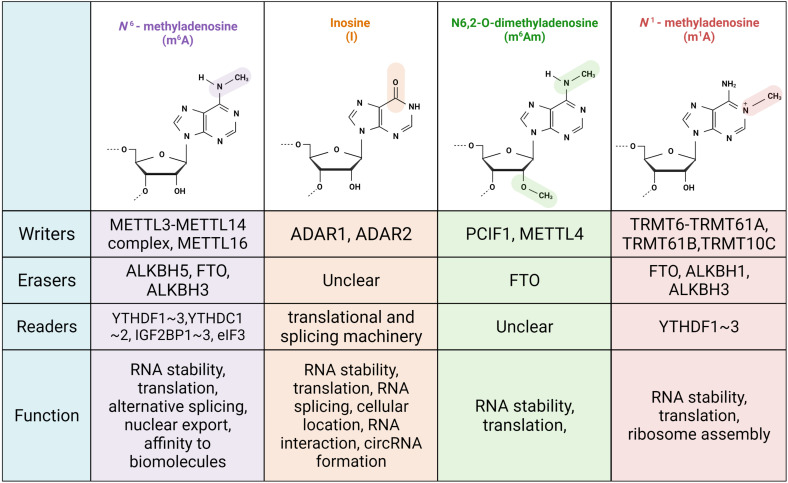
Schematic diagram of chemical formula, relative proteins and physiology function of common RNA modifications.

*N*^6^-Methyladenosine (m^6^A), the methylation occurring at the *N*^6^ of adenosine, is one of the most abundant mRNA modifications in eukaryotic cells (Perry et al., 1975; Wei et al., 1975). m^6^A is enriched in the 3′ untranslated region (3′UTR) and areas surrounding the coding sequence (CD) of mRNA with the typical consensus sequence DRACH (D=G, A, or U; R=G or A; H=A, C, or U) (Figure 1; Dominissini et al., 2012; Meyer et al., 2012). A series of proteins are involved in its formation, eradication, and function. The methyltransferase complex introduces methyl to adenosine co-transcriptionally, which includes METTL3, METTL14, and their co-factors, Wilms tumor 1-associated protein, VIRMA (KIAA1429), and RBM15. With the identification of demethylases, FTO and ALKBH5, m^6^A is believed to be a reversible and dynamic modification similar to histone and genome chemical modification (Jia et al., 2011; Zheng et al., 2013). Recently, Ueda et al. (2017) found that ALKBH3 demethylates m^6^A in tRNA rather than in mRNA or rRNA. m^6^A readers, including YTHDF1–3, YTHDC1–2, and the newly characterized IGF2BP1–3 and eIF3, recognize m^6^A and mediate downstream effects. YTHDF1 increases translation efficiency, whereas YTHDF2 decreases RNA stability (Wang et al., 2014, 2015). YTHDF3 assists in YTHDF1 and YTHDF2 activity (Li A. et al., 2017; Shi et al., 2017). YTHDC2 enhances translation efficiency and decreases RNA stability (Hsu et al., 2017). In addition, unlike other YTH proteins that mainly reside in the cytoplasm, YTHDC1 locates in the nucleus, regulating RNA splicing by recruiting pre-mRNA splicing factor SRSF3 and mRNA export by interacting with SRSF3 and nuclear-export adaptor protein (Xiao et al., 2016; Roundtree et al., 2017). eIF3 promotes cellular Cap-independent translation under stress conditions (Meyer et al., 2015; Zhou et al., 2015). Moreover, IGF2BPs interact with m^6^A and stabilize the modified transcripts under normal and stress conditions (Huang et al., 2018). In recent years, an increasing number of readers have been identified and demonstrated to be strongly associated with epi-transcriptome variation and transformations (Edens et al., 2019; Wu et al., 2019).

Owing to advances in high-throughput and high-resolution sequencing, m^6^A is found in almost every type of RNA, including mRNA, tRNA, rRNA, miRNA, lncRNA, circular RNA (circRNA), and snoRNA, and is virtually linked to major bioprocesses and normal cell development. The long non-coding RNA X-inactive specific transcript possesses 78 m^6^A residues, some of which induce transcriptional silencing via tethering to YTHDC1 (Patil et al., 2016). m^6^A in the stem-loop region of *MALAT1* increases its interaction with heterogeneous nuclear ribonucleoprotein C1/C2 (Zhou et al., 2016). m^6^A modifications in *lncRNA 1281* enable miRNA sequestration through RNA–RNA interactions (Yang et al., 2018). Oncogenic *lncRNA FAM225A* containing m^6^A residues has a longer lifetime and can sponge miRNA (Zheng et al., 2019). In contrast to the stabilization of m^6^A in *FAM225A*, residues in *lncRNA GAS5* induce RNA decay depending on YTHDF3 or YTHDF2 (Wang X. et al., 2019). METTL3 catalyzes pri-miRNA and promotes miRNA processing with the assistance of the RNA-binding proteins DGCR8 and hnRNPA2/B1 (Alarcon et al., 2015). *SNORD75* contains a region for METTL3 recognition, which regulates the splicing event of the host gene *GAS5* (Filippova et al., 2019). m^6^A in circRNA is an important regulator of RNA stability that relies on YTHDF2. However, whether stabilization or destabilization occurs depends on different cell states and types (Zhou et al., 2017). Yang et al. (2017) demonstrated that m^6^A in circRNA triggers translation initiation, in which the initiation factor eIF4G2 and m^6^A reader YTHDF3 play an essential role.

*N*^6^-Methyladenosine bioprocesses play a role in the regulation of disease states by manipulating RNA fate. However, some conclusions remain controversial. For leukemogenesis, compared with cord blood CD34 +, METTL3 is highly expressed in different AML cell lines and patient samples. Moreover, Vu et al. (2017) elucidated that METTL3 KD AML cells were more likely to undergo apoptosis and differentiation and exhibited lower pathogenicity. Mechanistically, overexpression of METTL3 in AML cell lines increased the abundance of m^6^A levels and triggered higher expression of c-MYC, PTEN, and BCL-2 (Vu et al., 2017). Another study also revealed that METTL3, the core of the methyltransferase complex, acts as a translation effector to regulate the development of AML (Barbieri et al., 2017). The expression of METTL14 is related to hematopoietic stem cells (HSCs) stemness and pluripotency, and METTL14 aberration contributes to malignant hematopoiesis. In addition, METTL14 plays an oncogenic role in AML by modifying downstream transcripts, such as *MYC* and *MYB*, and regulating their stability and translation efficiency (Weng et al., 2018). All these findings indicate that m^6^A is an oncogenic element. On the other hand, overexpression of FTO suppresses the propagation and transformation of AML cells and promotes leukemogenesis in mice (Li Z. et al., 2017). R-2HG directly diminishes the demethylase activity of FTO and, subsequently, increases the overall m^6^A level in R-2HG-sensitive AML cells, which destabilizes *MYC* and *CEBPA* transcripts. As a result, R-2HG treatment is prone to delay-sensitive AML propagation (Su et al., 2018). Newly identified specific FTO inhibitors; FB23, FB23-2, CS1, and CS2, are promising for anti-tumor therapy strategies (Huang et al., 2019; Su et al., 2020). The reason why both writers and erasers target the same transcripts (e.g., *MYC*) and contribute to the development of leukemia is probably related to the variation in function of m^6^A in specific regions of RNA. In other words, various readers may recognize different m^6^A in specific RNA and lead to diverse readouts. Therefore, it is urgently necessary to expand the toolkit to dissect individual m^6^A functions instead of ranges of modification.

#### Adenosine Deamination

Another prevalent RNA modification is adenosine-to-inosine editing, which is mediated by adenosine deaminase acting on the RNA (ADAR) family. Instead of pairing with uridine, inosine preferentially pairs with cytidine (Figure 1). There are three ADAR proteins in human cells: ADAR1, ADAR2, and ADAR3. These all have a C-terminal deaminase domain and an N-terminal dsRNA-binding domain. ADAR1 and ADAR2 are widely distributed among cells and can generate inosine, whereas ADAR3 has no deaminase activity and is mainly located in the brain (Bhakta and Tsukahara, 2020). The Z-binding domain in ADAR1 allows it to interact with Z-DNA and Z-RNA, and Z-RNA interaction promotes editing efficiency (Koeris et al., 2005). ADAR1 is the only member that can shuttle between the cytoplasm and nucleus, whereas ADAR2 is primarily present in the nucleus and nucleolus (Eckmann et al., 2001; Desterro et al., 2003).

Inosine in the open reading frame may not only cause amino acid substitutions but also halt ribosomes walking along the transcripts since inosines in the decoding center of the ribosome affect translation kinetics (Licht et al., 2019). A-to-I editing events in regions outside the coding sequence, such as the 3′UTR, intron, and 5′UTR, can also regulate gene expression by affecting small RNA interactions, RNA splicing, RNA stability, the cellular location of transcripts, and circRNA formation. Rat ADAR2 self-regulates by modulating its alternative splicing. Introducing AI at the 3′splice junction results in an additional 47 nucleotides in the mature *rAdar2* mRNA. As a result, frameshift mutations delete the RNA-binding and deaminase domains (Laurencikiene et al., 2006). Solomon et al. (2013) suggested that the regulation of trans-factors involved in the splicing machinery caused global splicing pattern variation in ADAR KD cells. Circular RNAs (circRNA), a family of non-coding RNA, are generated by the back-splicing of coding transcripts. Circulation of circRNAs depends on the base-pairing of inverted repeat sequences, such as *Alu*, or RNA-binding proteins, such as fused in sarcoma (FUS). Long and stable *Alu* acts as a platform for recruiting multiple ADARs, then ADARs edit at nearby short hairpins. Editing at reverse complementary sequences, which brackets a circRNA, melts the stem structure and ultimately represses circRNA formation (Daniel et al., 2014). Helicase HDX9 has a strong inclination to interact with the *Alu* sequence and downregulate circRNA generation by disrupting the double-stranded region. HDX9 also binds to ADARp150 in an RNA-independent manner (Aktas et al., 2017). U:I or I:U induced by ADARs in pre-miRNA destroys the overall tertiary structure and alters the dsRNA structure, subsequently undermining the interaction with RNA-binding proteins, such as Drosha; an RNase related to miRNA biogenesis (Yang et al., 2006). Interestingly, catalytic domain mutations in ADAR affect miRNA genesis similarly since ADAR competitively binds to pre-miRNA against Drosha (Borchert et al., 2009). When ADARs edit pre-miRNA precursors, TSN, the main catalytic component of RISC, specifically destroys it (Scadden, 2005). Editing at the seed region of miRNA results in novel targeted RNA due to variations in the targeted sequences. Therefore, there are extensive interactions between RNA editing and interference (RNAi) pathways.

A-to-I editing may lead to inflammatory disorders and negative effect in many cancers. For example, splicing alteration originating from A-to-I editing partially contributes to human malignant brain tumors because the Q/R transition generates disordered *GluR* isoforms (Schoft et al., 2007). Apart from neural disorders, there are many other malignant transformations associated with the abnormal expression of ADARs and aberrant alternative splicing, such as Chronic Myeloid Leukemia, dyschromatosis symmetrica hereditaria, and hepatocarcinoma (Ansai et al., 2016).

#### *N*^6^,*O*2′-Dimethyladenosine (m^6^Am)

The di-methylated nucleoside m^6^Am is mainly located in the second position linked to the triphosphate bridge to 7-methylguanosine (m7G) (Figure 1). Several m^6^Am modifications are in the third position, and their biogenesis and physiological functions are largely unknown. m^6^Am and Am contribute to 70% of m7G (5′) ppp (5′) Nm and m7G (5′) ppp (5′) Nm. Mauer et al. (2017) first believed that m^6^Am was a reversible modification, as they found that FTO, an m^6^A eraser, had demethylation activity toward m^6^Am. They also illustrated that m^6^Am-initiated RNA in FTO KD cells had a longer lifetime owing to resistance to the decapping protein DCP2. Likewise, Boulias et al. (2019) reported that m^6^Am increased RNA stability, whose degradation relied on DCP2. Further study of the direct substrates of FTO comprehensively revealed that FTO targeted multiple RNA species besides messenger RNA, including small nuclear RNA (snRNA) and tRNA. However, substrate preference was found to be closely related to FTO location; FTO in cytoplasm prioritizes demethylating m^6^Am, while the nuclear version tends to affect m^6^A (Wei et al., 2018). FTO also manipulates m^6^Am and m^6^A levels in snRNAs (Mauer et al., 2019). Furthermore, crystal structure analysis showed that FTO has the same tendency toward m^6^A and m^6^Am in individual RNA, ignoring the ribosomal ring (Zhang et al., 2019). Mauer et al. (2019), who persistently labeled m^6^Am as the major target of FTO, found that FTO altered the biogenesis process of Sm-snRNA, including the involvement of U1, U2, and U4. snRNA is generated by RNA polymerase II (RNAPII) and incorporated into the spliceosome through a set of processing events in which FTO is responsible for the transformation of two methylated states; m1 and m2 (Mauer et al., 2019). Several groups demonstrated Phosphorylated C-terminal domain (CTD)-interacting factor 1 (PCIF1) acted as a writer of m^6^Am almost at the same time, but the m^6^Am physiological function remains controversial and requires further investigation (Boulias et al., 2019; Sendinc et al., 2019). Cap-specific adenosine-*N*^6^-methyltransferase (CAPAM; also known as PCIF1) methylates m7GpppAm in an S-adenosylmethionine-dependent manner, in which MTase and the helical domain form a pocket to recognize the m7G cap. Given that the N-terminal WW domain of CAPAM has a high affinity for the Ser5-phosphorylated CTD of RNAPII, CAPAM introduces m^6^Am to a nascent mRNA chain co-transcriptionally (Akichika et al., 2019). In contrast to the previous discovery that m^6^Am promotes transcriptional stabilization, Akichika et al. (2019) illustrated that CAPAM KO human cells undermine translation efficiency and slightly decrease RNA levels according to a ribosome profiling assay and RNA-seq. In human melanoma cell lines, reporter assays and tandem mass tag proteomic quantification showed that m^6^Am tends to be an inhibitory signal for cap-dependent translation and has no internal crosstalk with m^6^A (Akichika et al., 2019). Of note, a recent study showed that METTL4 introduced internal m^6^Am to snRNA then modulated the RNA splicing process (Chen et al., 2020). Several factors contribute to the diverse conclusions regarding m^6^Am, as follows: (1) the stress and cell state conditions of these studies differ, thus, m^6^Am predominantly affects translation or RNA stability; (2) m^6^Am is a cell type-specific epi-transcriptomic marker; and (3) diverse sequence motifs and unidentified readers result in uncertain annotations.

#### *N*^1^-Methyladenosine

*N*^1^-methyladenosine, which was identified in the 1960s, is more enriched in tRNA and rRNA than in mRNA (Figure 1). This type of modified nucleotide exists in various organisms, such as plants, mammals, bacteria, protists, and even multiple tissues and physiological fluids (Droogmans et al., 2003). Position 58 in the TΨC loop of many eukaryotic tRNAs is conservatively methylated. Gcd10p (TRMT6)-Gcd14p (TRMT61A) is the first identified methyltransferase that is responsible for the formation of m^1^A58 at Met-tRNA_i_^Met^. Position 58 of the initiator tRNA plays a central role in maintaining the tRNA tertiary structure. Gcd10p mutation hinders Met-tRNA_i_^Met^ processing and triggers Met-tRNA_i_^Met^ polyadenylation, but does not affect its own pre-mRNA synthesis and other tRNA maturation (Anderson et al., 1998). m^1^A-containing tRNA has a longer lifetime, which also results from increased hydrophobicity and increased positive charge around the methyl group. In addition to tRNA, m^1^A modification of mRNA and lncRNA is catalyzed by TRMT6 and TRMT61. m^1^A modification in messenger RNA is inextricably linked to its translation capability. With the overexpression of TRMT6 and TRMT61 in 293T cells, m^1^A-modified mRNA transitioned from the ribosome-heavy to ribosome-light fraction. When tRNA binds to mRNA, m^1^A residues in the triple codon impede base pairing and render subsequent early termination of translation and undermined translation efficiency (Safra et al., 2017). For mitochondrial tRNAs, such as tRNA^Leu^^(*UUR*)^, bovine tRNA^Ser (UCN)^, and bovine tRNA^Glu^, TRMT61B acts as a mitochondrion-specific methyltransferase at position 58 in the TΨC loop. TRMT61B also regulates m^1^A947 formation in mitochondrial 16s rRNA, which is critical for the mito-ribosome structure and translation of mtDNA-encoding proteins (Bar-Yaacov et al., 2016). Li found that TRMT61B could catalyze m^1^A on mt-mRNA, such as *MT-CO1*, *MT-CO2*, *MT-CO3*, and *ND4*. The methylation modification of CDs of mitochondrial transcripts inhibits translation. When knocking down TRMT61B, on the one hand, upregulated mt-mRNA translation increases its protein abundance; on the other hand, the decreased methylation levels of mt-rRNA and mt-tRNA undermine protein synthesis. Therefore, the physiological effects of mitochondrial m^1^A undergo sophisticated regulation (Li X. et al., 2017). TRMT10C-SDR5C1 also introduces m^1^A, in which TRMT10C requires SDR5C1 to be active as a methyltransferase (Vilardo et al., 2012). Rrp8 (NML1 in humans) and BMT2 regulate the formation of m^1^A in rRNA, thereby controlling ribosome assembly (Sharma et al., 2013, 2018; Waku et al., 2016). Many demethylases of m^1^A have been characterized successively. Knocking down ALKBH1 principally mimics the effect of overexpressing TRMT6 and TRMT61A, boosting both initiation and elongation progress with the recognition of EF1a (Liu et al., 2016). ALKBH3 catalyzes not only tRNA but also mRNA (Ueda et al., 2017). Moreover, ALKB family proteins also participate in mRNA homeostasis. By directly analyzing FTO substrates with CLIP profiling, FTO was found to bind to m^1^A58 and showed a similar role to ALKBH1. YTHDF3, which is a reader that recognizes m^6^A, also attaches to m^1^A and induces mRNA decay, for example, that of *IGF1R*. In trophoblast cells, the degradation of *IGF1R* mediated by YTHDF3 leads to the repression of the IGF signaling pathway, thus inhibiting downstream matrix metallopeptidase 9 signaling and preventing the invasion of trophoblast cells. This discovery may provide a new target for the treatment of pregnancy disorders (Zheng et al., 2020).

In addition to the correlation between m^1^A and physiological function, m^6^Am also contributes to disease status. m^1^A writer NML1 is associated with abnormal obesity. In cells with NML1 deletion, inhibition of the 60S large unit assembly results in enhanced affinity between RPL11 and P53 and constantly activates the P53 pathway, triggering cell cycle arrest and cell apoptosis (Waku et al., 2016). ALKBH3 overexpressing cells become more sensitive to angiotensin (ANG), and ANG can cut the anti-codon region of tRNA and produce tDRs, which promote ribosome reassembly and inhibit cell apoptosis by combining with Cyt C (Chen et al., 2019). Mitochondrial DNA mutation (m8344A>G) in myoclonus epilepsy and ragged-red fibers leads to the absence of tRNA^Lys^ A58 modification, which diminishes the interaction between translation effectors EF-Tu and tRNA^Lys^, undermines nascent peptide synthesis, and generates abnormal protein (Richter et al., 2018). m^1^A is closely related to the expression of oncogenic proteins and genetic mutations according to bio-information analysis of the data of gastrointestinal cancer patients from The Cancer Genome Atlas database (Zhao et al., 2019).

## Engineering of RNase-Defective Cas13 (dCas13) Fused With RNA Modification Enzymes

### A-to-I Editing

**FIGURE 2:**
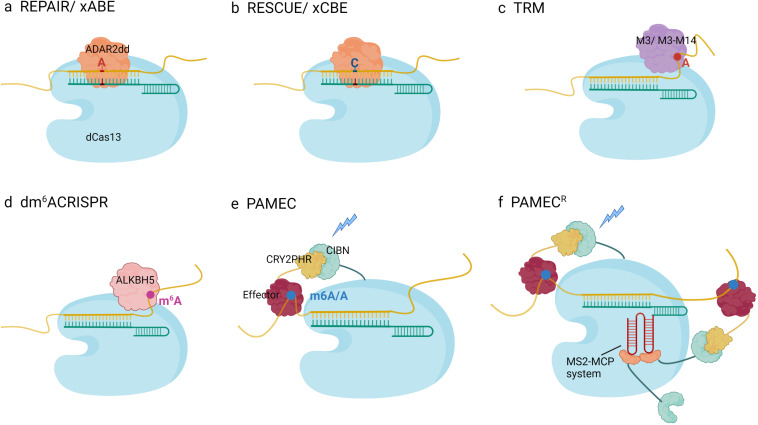
Schematic diagram of RNA modification engineering platforms: **(A)** REPAIR/xABE (A-to-I editing platform): Specifically designed CrRNAs base-pair with RNA targets and recruit dCas13-ADAR2dd to the dsRNA, which deaminates adenosines to inosines in the dsRNA region. C-A mismatch in editing sites enhances the editing specificity. **(B)** RESCUE/xCBE (C-to-U editing platform): dCas13 is fused to evolutionary ADAR2dd, which performs increased cytidine deamination activity. With the guidance of CrRNA, dCas13-ADAR2dd mediates A-to-U transformation in the dsRNA. A mismatched uridine in the CrRNA opposite the target cytidine enhances the editing reaction. **(C)** The targeted RNA methylation (TRM) system: dCas13b fusions to modified methyltransferase domains, such as METTL3ΔZF (M3) removing zinc finger RNA binding motif or constructed protein consisted with M3 and METTL3-interacting domain of METTL14 can selectively and efficiently install m^6^A in targeted adenosine. **(D)** dm^6^ACRISPR system: The targeted demethylation system is consist of a catalytically inactive Cas13b, full-length of demethylase ALKBH5 and gRNA, which enable to demethylate either close or remote m^6^A modifications. **(E)** Photoactivatable RNA m^6^A editing system using CRISPR-dCas13 (PAMEC): PAMEC harnesses CIBN-CRY2, a robust light-dependent protein–protein interaction system. Under the explosion of blue light, the RNA anchor probe including CIBN and dCas13b and the effector probe containing RNA editing effector and CRY2 tight together and form a functional unit for m^6^A installation and erasure. **(F)** PAMECR: RNA–protein interaction system, MS2-MCP, is further introduced to primary PAMEC platform, which ensures manipulation of multiple transcripts robustly and simultaneously.

To alter specific target transcripts, several techniques have been developed to deliver the catalytic domain of ADAR or recruit endogenous ADAR to an adenosine site, which overcomes the deleterious effect of ADAR overexpression. Given that PspCas13 silence targeted RNAs efficiently and specifically, Cox introduced mutations to the HEPN domain and generated dPspCas13b lacking nuclease activity. After fusing the ADAR effector with several amino acid linkers, hyper-activating versions of ADAR1 (E1008Q) or ADAR2 (E488Q) were adapted to increase productivity (Cox et al., 2017). To optimize this system, the authors tested the length of CrRNA and linker characteristics and found that ADAR2 (E488Q) had higher compatibility with CrRNA length and showed more robust editing capability, whereas ADAR1 (E1008Q) depended on a much longer CrRNA, and linker construction was not that important for improving efficiency. Based on the above optimized strategy, the first epi-transcriptome editing platform utilizing dCas13b was engineered and termed RNA Editing for Programmable A to I Replacement version 1 (REPAIRv1) (Figure 2A). However, the editing rate of endogenous transcripts (28% in *PPIB*) and correction of disease-related mutations (35% correction of *AVPR2* and 23% correction of *FANCC*) remained inefficient; even the specificity was subpar. To maintain the core structure of REPAIRv1, a mismatch in the editing site (C-A) of the base-pairing region was introduced, and substitution in the RNA-binding region of ADAR was generated to lower the affinity of ADAR and gain more specificity. Compared with REPAIRv1, REPAIRv2 (dCas13b-ADAR2 (E488Q/T375G) exhibited a lower off-target rate, but the editing efficiency slightly reduced (23.7% for *KRAS* and 13% for *PPIB*) (Cox et al., 2017). Similar to REPAIRv2, Xu et al. fused dCas13x.1 with high-fidelity ADAR2dd (with E488Q/T375G) to generate A-to-I RNA base editors (termed ‘xABE,’ 1195aa). Truncated dCas13x.1 was harnessed to minimize the editor size for efficient AAV packaging (termed ‘mxABE,’ 865aa) (Figure 2A). Furthermore, they found editing position from 15 to 25 nt on the 50-nt CrRNA yielded higher editing efficiency. Both xABE and mxABE achieved modest editing toward endogenous transcripts (Xu et al., 2021). There are several single-base A-to-I editing machines that use different strategies, such as chemical elements or CRISPR-inspired systems. ADAR substrates, such as *GluR2*, and RNA–protein binding platforms, such as the MS2-MCP system, are adapted to recruit endogenous ADAR. Merkle et al. (2019) constructed antisense oligonucleotides (ASOs) with 2′-*O*-methylation and phosphorylated chemical modification, termed RESTORE, to recruit endogenous ADAR isoforms to an editing site, thus, could transform A to I without perturbing natural editing homeostasis. Chemical modification, mismatch in editing sites, and optimization of engineered ASOs guarantee a low off-target rate, recruiting yield, and delivery efficiency. Nevertheless, there remain several limitations to be addressed. First, chemical decoration possesses immunogenicity and can evoke the immune response. Second, IFN-α plays a critical role in promoting transformation events; however, some side-effects in hematological system, nervous system and endocrine system may increase the risk of clinical application (Zdilar et al., 2000; Tomer et al., 2007; Wada et al., 2009). Leveraging endogenous ADAR for programmable editing of RNA overcomes the immunogenicity of chemically modified oligonucleotides; its nucleotide conversion only depends on 111–191 nt ADAR-recruiting RNAs (arRNAs) in light of CrRNA recruiting endogenous ADAR proteins and can correct pathogenic mutations in constructed mammalian cells and multiple human primary cells without affecting natural editing homeostasis and cellular gene expression (Qu et al., 2019). As there is an imbalance between accuracy and efficiency, more factors must be considered. Combining the dCas13 system with chemical approaches to remodel adenosine is of great importance. On the one hand, utilizing a specially designed or RNA–protein interaction platform, such as the MS2-MCP system, is a good way to increase effector concentration in editing sites and improve editing capacity. On the other hand, it is necessary to improve dCas13, which possesses controllable and temporary characteristics, for basic researches and further clinical applications.

### C-to-U Editing

Inspired by the A-to-I editing scaffold, a C-to-U editing machine was developed. Such a C-to-U RNA base editor largely expands the toolkit related to repairing disease-causing mutations at the RNA level. Genomic cytidine editor consisting of nCas9 and cytidine deaminase APOBEC1 exhibits relatively high off-targets and low site specificity. Therefore existing APOBEC1 is less likely to be engaged in single-base C-to-U editors. Given that the ADAR adenosine deaminase domain is homologous to the *Escherichia coli* cytidine deaminase, Abudayyeh et al. (2019) suggested that ADAR could be adapted to function as cytidine deaminase. After 16 rounds of rational mutation and late fusion with dRanCas13b (Cas13b from *Riemerella anatipestifer*), ADAR2dd became the catalytic domain of RNA Editing for Specific C-to-U Exchange (RESCUE), a new generation epi-transcriptome editing platform (Figure 2B). Previous studies have found that in the luciferase restoration assay introducing C or U mismatch at the editing site and 30 nt CrRNA, RESCUE exhibits the most robust editing activity. In HEK293FT cells, RESCUE works efficiently toward endogenous transcripts, such as *CTNNB1*, a critical phosphorylation residue involved in STAT and Wnt/β-catenin pathways, which results in the activation of signaling and cell growth stimulation. Utilizing ADAR2dd raises the challenge of the A-to-I off-target effect in addition to substantial C-to-U off-targeting. Previous studies have mutated the RNA-targeting residues in ADAR2dd to improve specificity and introduced S375A to advanced RESCUE (RESCUE-S) (Abudayyeh et al., 2019). Based on the above evolved ADAR2dd, xCBE and smaller mxCBE utilized dCasx.1 achieved more robust C-to-U editing than RESCUE-S in mammalian cells (Xu et al., 2021; Figure 2B). In addition to the C-to-U editing platform, the range of RNA editing available has greatly expanded.

### RNA *N*^6^-Methyladenosine Editing

After the application of the CRISPR-Cas13 system in mRNA deamination, it was subsequently adapted as a machine to study the molecular basis of m^6^A-mediated RNA regulation at individual transcripts. By replacing ADAR2dd in the REPAIR platform with the N-terminal domain of YTHDF1 or the CCR4-NOT binding domain of YTHDF2, dCas13b can be used to study specific reader’s functions on single transcripts in an m^6^A-independent manner, which ignores the cooperation and antagonism of various readers and protein complexes. The reporter targeting assay implies that YTHDF1 and YTHDF2 are consistent with the reported characteristics of translational promotion and RNA decay mediation (Rauch et al., 2018). Another platform, called the CRISPR-Cas-inspired RNA targeting system (CIRT) is inspired by the CRISPR-Cas system and uses a total human part of single-strand RNA binding protein and stem-loop RNA binding unit to decrease immunogenicity. By linking these proteins with the catalytic domain of the effector, such as the translation-regulating region of YTHDF1 and the RNA decay-mediating domain of YTHDF2, CIRTs can regulate various RNA-related physiological processes (Rauch et al., 2019). Even so, these only provide the possibility to study the function of m^6^A-containing transcripts, whereas the function of single m^6^A modification in specific transcription remains to be determined. Fusing either “Writer” or “Eraser” with the CRISPER-dCas9 system edits the single site m^6^A in different RNA regions, such as the 3′-UTR of *ACTB*, 5′UTR of heat-shock protein, and even non-coding RNA *MALAT1* (Liu et al., 2019; Rau et al., 2019).

The targeted RNA methylation (TRM) system utilizes the catalytic domain of methyl transferase METTL3 or fusion version with METTL14 linked to dCas13b, and the zinc finger of METTL3 in TRM is deleted to enhance the binding affinity between dCas13b and RNA (Figure 2C). By fusing with different location tags, TRM can manipulate m^6^A modification with different RNA states and cellular locations and trigger various readouts, ignoring the physiological function of targeted proteins, such as co-export-associated proteins. Among the four combinations with two locational tags, dCas13-M3nls and dCas13-M3M14nls showed the best performance. These two platforms are capable of multiplying the m^6^A level in specific transcripts, such as *ACTB*, *GAPDH*, *FOXM1*, and *SOX2*, and these editing events are selective on targeted residues with the assistance of CrRNA. A 30 nucleotide length of CrRNA ended 8–15 nucleotides upstream of the targeted site, ensuring editing specificity. dCas13-M3, dCas13-M3M14, and M3M14-dCas9 displayed similar performance in on-target efficiency; however, dCas13-M3 lacking the RNA-binding protein METTL14 exhibited greater specificity (Wilson et al., 2020). In contrast to TRM using methyltransferase, a previous study showed that by constructing PspCas13b and m^6^A demethylase ALKBH5 using a flexible linker, dm^6^ACRISPR increased mRNA stability of *CYB5A* and promoted the translation efficiency of β-catenin with limited off-target effects (Figure 2D). Selective qPCR showed that dm^6^ACRISPR had the highest ability to demethylate modified endogenous genes (*ACTB* 80% and *CTNNB1* 90%) with a low off-target possibility. In addition, there was no additive effect when using sets of CrRNA to target the same transcripts (Li et al., 2020). Nonetheless, all the experiments in the study were conducted on selected RNA molecules with only one modified site, thus, it was not known whether this system could alter more than one m^6^A in a transcript.

None of the methods discussed above achieve spatiotemporal regulation of m^6^A modification. Given that the light-sensitive protein CBIN and its adaptor CRY2 are tightly packed together under blue light exposure, dCas13 fused with CBIN and CRY2 linked to the full-length FTO (PAMEC1) or essential domain of the METTL3/METTL14 heterodimer (PAMEC2) will also be located close to each other and catalyze the substrate under blue light (Figure 2E). With the presence or absence of blue light, cellular m^6^A levels vary spatially and temporally. PAMEC1 can demethylate m^6^A sites in *MALAT1*, while PAMEC2 methylates adenosine in *ACTB* and *TPT1*. Moreover, advanced PAMEC^R^ with the MS2-MCP RNA–protein interaction system enables the recruitment of more effectors to the targeting site (Figure 2F). PAMEC^R^ not only exhibits higher editing efficiency but also makes multiple editing events with sets of CrRNA possible. When blue light is turned on, PAMEC^R^ works, but once the light variation induced by PAMEC^R^ is turned off, it decreases to the baseline gradually and becomes equivalent to the state before irradiation. Therefore, PAMEC^R^ is a controllable scaffold. Moreover, previous studies have utilized upconversion nanoparticles, which emit blue light after excitation by the near infrared spectrum, to overcome the poor penetration of short-wavelength light and realize m^6^A manipulation *in vivo* (Zhao et al., 2020).

There is still considerable scope to investigate the use of such an RNA editing programmable tool. As the consequence of overall overexpression or interference of a key protein is univocal and integrated, it is not currently known whether various m^6^A modifications in individual transcripts work in a synergistic, antagonistic, or dynamic balance. Additionally, whether m^6^A in a particular region of different transcripts plays the same role is not currently known.

### m^6^Am Editing

Although m^6^Am was first identified 40 years ago, associated projects have been extremely lacking until recently. There have been no reports on m^6^Am programming using different toolkits. There are several reasons for this: Firstly, primary m^6^A antibody-based sequence approaches, a low-resolution mapping technology that cannot substantially distinguish m^6^Am from m^6^A, mistakenly assigned several m^6^Am-linked physiological effects to m^6^A. Many studies regarding FTO have demonstrated that m^6^A and m^6^Am are involved in the neuronal system, immune response, tumor genesis, and malignancy. Nevertheless, these findings are based on m^6^A- and m^6^Am-seq or m^6^A antibodies. A novel high-throughput mapping method; m^6^Am-Exo-Seq, performed in an exonuclease-dependent manner, is m^6^Am-specific. Using this technique, FTO was endowed with m^6^Am demethylase activity, which impaired the metastasis and chemoresistance of colorectal cancer stem cells (Sendinc et al., 2019). Secondly, it is commonly believed that the m^6^Am level is extremely low. However, RNA-MS analysis demonstrated that m^6^Am modification in human mRNAs is much more abundant than previously thought, and m^6^Am is a highly conserved modification in all vertebrate organisms and contributes to various physiological and pathological processes, such as the oxidative stress response, Kaposi’s sarcoma-associated herpesvirus infection, and tumor genesis and malignance (Tan et al., 2018). Moreover, m^6^Am is closely linked to RNA fate, stability, and translatability. Therefore, it is important to modulate the m^6^Am modification of RNA in a directional manner.

## Application of dCas13-RNA Modification Enzyme Fusion Constructs in Cell Biology and Diseases

### Site-Specific Regulation of RNA Modification

Targeted RNA methylation increased the methylation level of *ACTB* A1216 by two-to-five times, and the mRNA abundance of *ACTB* in HEK293FT cells also decreased significantly, in line with a previous study of m^6^A in YTHDF2-inducing mRNA decay (dCas13-M3nls with 35–42% and dCas13-M3M14nes with 45–70%). Although *GAPDH* A690 modification increased, the RNA levels did not change. As previous studies did not test the protein level and other variations, the role of *GAPDH* m^6^A690 was uncertain. In addition, targeting *BRD8* and *ZNF638* by nucleus-located version dCas13-M3nls modulates alternative splicing of these pre-mRNAs and controls exon21 exclusion or inclusion (Wilson et al., 2020). Abolishing the m^6^A site of *CYB5A* by dm^6^ACRISPR resulted in increased RNA levels, which demonstrated that m^6^A in *CYB5A* modulated RNA stability. dm^6^ACRISPR targeting three residues in the 5′UTR of *CTNNB15* increases both RNA level (less than two-fold) and protein level (around three-fold). m^6^A residues in the 5′UTR of *CTNNB15* may downregulate RNA stability and translation capability, which contrasts with the previous finding that m^6^A in the 5′UTR promotes translation by interacting with the translation initiation factor. PAMECs also manipulate RNA fate. PAMEC-2 adds a methyl group to the 3′-UTR of *TPT1*, giving rise to RNA decay (Li et al., 2020).

### Cell Fate Engineering

Studies have performed C-to-U editing in the serine residue of β-catenin, a critical factor of STAT and Wnt/β-catenin pathways, to introduce missense mutations. Phosphorylation site abrogation inhibited the ubiquitination and degradation of β-catenin, subsequently activating the STAT and Wnt/β-catenin pathways and accelerating the growth of HEK293FT cells and HUVECs (Abudayyeh et al., 2019). In bone marrow mesenchymal stem cells, demethylation of m^6^A in *PTH1R* mRNA through PAMEC^R^ undermines translation efficiency without changing RNA stability and blocks osteogenic differentiation (Zhao et al., 2020).

### Development and Diseases

Multiple platforms have been engineered to detect pathogens, the majority of which apply collateral nuclease activity. SHERLOCKv1 and advanced SHELOCKv2 have been used to rapidly detect dengue fever virus and ZIKV and can also identify bacterial isolates, antibiotic resistance genes, human DNA genotypes, and cancer-associated mutations with single-base resolution (Gootenberg et al., 2017, 2018). The newly developed platform Combinatorial Arrayed Reactions for Multiplexed Evaluation of Nucleic acids-Cas13 can detect severe acute respiratory syndrome coronavirus 2, various human immunodeficiency virus genetic mutations accounting for drug resistance, and different types of influenza A in a high-throughput manner (Ackerman et al., 2020). With the guidance of CrRNA, Cas13 can reduce harmful exogenous genetic materials. The CARVER and PAC-MEN strategies both perform well in degrading virus RNA and inhibit virus infection, while the former employs PspCas13b and the latter harnesses smaller Cas13d (Freije et al., 2019; Abbott et al., 2020). HUDSON and SHERLOCK can be used to assess the therapeutic effects (Myhrvold et al., 2018). Apart from virus detection and targeting, Cas13 system can be harnessed to correct disease-related mutations and treat different types of disorders. Knocking down *mPtbp1* by CasRx is sufficient to transform Muller glia cells into functional retinal ganglion cells, which partially restores the visual responses in mouse model with permanent vision loss. Moreover, it also induces astrocytes in striatum into dopamine neurons and reduces motor dysfunction in the PD model mice (Zhou et al., 2020). Targeting exon 10 of *MAPT* mRNA by dCasRX results in the exclusion of exon 10, which destroys the homeostasis between two isoforms related to occurrence of neurodegenerative disease. REPAIRv1 was applied to correct the G-A pair substitution of *AVPR2*, W293X in X-linked nephrogenic diabetes insipidus, *FANCC* W506X in Fanconi anemia, and 34 other disease-relevant mutations in a variety of disorders (Cox et al., 2017). However, the editing efficiency was extremely low. REPAIRv2 is able to correct nonsense mutation of transmembrane conductance regulator (*CFTR*) gene in different cell types, the pathogenic factor of cystic fibrosis (Melfi et al., 2020). C-to-U editing platform is a useful tool for editing 24 synthetic disease-relevant mutation targets from ClinVar database with the editing rates up to 42% (Abudayyeh et al., 2019). Erasing m^6^A residues in *EGFR* and *MYC* dramatically undermined the proliferation of Hela cells due to the decreased affinity to YTHDF1 as well as the decreased YTHDF1-evaluated mRNA translation level (Li et al., 2020).

### Imaging

dCas13a and its derivatives enable RNA pull-down to study RNA–protein interactions, imaging via reconstitution with split fluorophores, translational modulation, RNA base editing, epi-transcriptome perturbation, splicing modulation, or induction of apoptosis based on RNA expression levels, which are useful for studying specific cell populations or killing cancerous cells. Abudayyeh et al. (2017) generated an *in vivo* platform called dCas13a-NF to image RNA and tracked RNA to stress granules, in which a negative-feedback system relying on zinc finger self-target and KRAB domain repression reduced the background signal from off-targeting. The above system could significantly interact with *ACTB* mRNA overlapping with fluorescent *in situ* hybridization (FISH) signals and track *ACTB* transcripts with stress granules by visualizing the stress granule marker G3BP1 (Abudayyeh et al., 2017). CRISPR live-cell FISH simultaneously visualizes DNA and RNA in live cells by harnessing dCas9, dCasRx, biotin, or fluorescence-marked gRNA. By co-delivering gMS2 and dCas9/gLacO into U2OS expressing dCas13d, Wang H. et al. (2019) successfully tracked both the DNA locus and RNA transcripts after doxycycline induction. Other groups have also revealed the same application (Yang et al., 2019; Davis and O’Connell, 2020). Contradictorily, Yang et al. (2019) utilized the most efficient dPspCas13b rather than dCasRx, as they observed abnormal co-localization of target transcripts of dCasRx and demonstrated the promising signal induced by low specificity. In addition to DNA and RNA labeling, the combination of the dCas13b and MS2-MCP strategies allowed the simultaneous imaging of more than one transcript (Yang et al., 2019). Therefore, the engineering of a versatile image approach is promising for the study of epigenetics and to expand our understanding of central dogma and nuclear activities, such as by visualizing how epigenetic modifications are generated and erased dynamically, exploring genetic repair events, determining what materials have changed when disease initiates, understanding malignant transformations, and so forth.

## Major Concerns and Optimization Strategies

### The Specificity of Epigenetic Engineering Platforms

**FIGURE 3:**
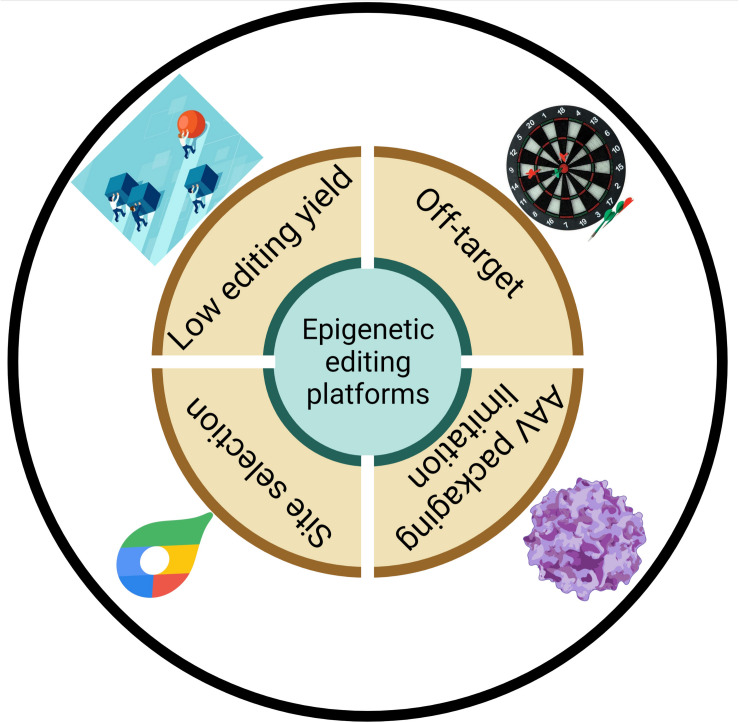
Schematic diagram of potential limitations of epigenetic editing platforms.

When discussing the CRISPR-Cas system, the most obvious repercussion to consider is widespread off-target effects (Figure 3). It is also a major concern for the CRISPR-Cas system in terms of further clinical applications. Two factors are responsible for off-target editing: the CrRNA and effectors. On one hand, the length and sequence construction of CrRNA spacers is related to the off-target capability. The REPAIR system required a 50-nt length of spacers to achieve the highest editing, but this also increased the probability of targeting at an improper location. Due to the extension of the double-strand region formed by complementary base pairing, other adenosines in this double-strand region may be edited. Unlike the REPAIR platform, RESCUE harnesses a shorter 30-nt length of spacers to lower the off-target rate. Furthermore, spacer sequence also play an important role in editing accuracy. For example, cytidine in spacers mispairing with adenosine narrows the editing site while mismatches in the middle seed region of spacers cause dramatically defects in on-target editing events (Cox et al., 2017; Abudayyeh et al., 2019). Moreover, the direct-repeats (DR) region of CrRNAs was shown to have the ability to recruit endogenous or expressed ADAR, which led to some degree of background editing (Marina et al., 2020). As for epigenetic engineering platforms, another possible reason for off-targets is that artificial effectors can catalyze undesirable transcripts which are spatially closed. David Cox elucidated that ADAR2dd triggered off-targets independent of dCas13 targeting. Therefore, destabilizing effector–RNA binding are available for selectively decreasing off-target events. Lowering the interaction of RNA and the ADAR effector by replacing T with G in the 375 residue of ADAR2 (E488Q) dramatically increased editing specificity, with a reduction from 18,385 to 20 transcriptome-wide off-targets in luciferase reporter assay (Cox et al., 2017). However, Vogel et al. (2018) and Marina et al. (2020) elucidated that there still was massive global off-target editing caused by overexpression of editases. Similarly, introducing S375A in the C-to-U editing system reduces the off-target rate to 10% without altering on-target efficiency (Abudayyeh et al., 2019). Compared with dCas13-M3M14nes, dCas13-M3nls lacking the RNA binding unit of methyltransferase complex did not change overall cellular m^6^A abundance without the guidance of CrRNA (Wilson et al., 2020).

### Site Selection for RNA Modification

It is important for transcriptome editing platforms to recognize the individual sites of targeted RNA. Cellular RNA naturally forms a tertiary structure, and Cas13 prefers to cut the single-strand region compared with the double-stranded region according to accessibility. However, editing preference depends on particular effectors, such as ADAR in the REPAIR deaminizing double-strand region. Moreover, exchanging the original A-U base pairing with A-C mismatch improves the selectivity toward adenosine and mutating ADAR protein to the hyper-activation version, further advancing the editing efficiency (Cox et al., 2017). Based on the substrate preference of ADAR, the C-to-U alteration platform applied mutant ADAR2dd instead of existing cytidine deaminase to ensure editing at the anticipated site (Abudayyeh et al., 2019). Not all adenosine can be methylated, and m^6^A is inclined to occur at the DRACH motif. METTL3 and METTL14 in TRM narrow the scope of the modified site, and CrRNA ending at 8–15 nucleotides upstream of targeted adenosine enables the accurate catalysis of individual adenosine according to the Me-RIP sequence (Wilson et al., 2020).

### Low Editing Efficiency

Editing efficiency is coordinated to the therapeutic effect. However, low editing yield is one of the most noticeable drawbacks of epigenetic engineering platform based on Cas13. Compared with other A-to-I editing platforms such as SNAP-ADARs with editing rate of 50–90% for endogenous gene, REPAIRv1 and advanced REPAIRV2 perform with an extremely low on-target rate (<35%) (Cox et al., 2017; Vogel et al., 2018; Vogel and Stafforst, 2019; Table 2). Likewise, A-to-I editing machine (xABE) based on smaller Cas13x.1 exhibited much lower editing efficiency than SNAP-ADARs (Xu et al., 2021; Figure 3). Given that A-to-I editing system did not exhibit the key strengths, some scientists suspected that the RNA-targeting CRISPR/Cas platforms will be superior to other RNA manipulating strategies (Vogel and Stafforst, 2019). RESCUE performed about 20% on-target rate while the on-target efficiency of xCBE was about 48% (Abudayyeh et al., 2019; Xu et al., 2021; Table 2). In terms of m^6^A editing, the variation of demethylation and methylation after PAMEC treatment was about 50%. However, advanced PAMEC increased m^6^A level by 4- to 4.5-fold (Zhao et al., 2020; Table 2). dCas13–M3nls and dCas13–M3M14nes exhibited promising methylation abilities toward *GAPDH* and *FOXM1* (Wilson et al., 2020). dm^6^ACRISPR rendered 80–90% demethylation efficiency (Li et al., 2020; Table 2).

**TABLE 2:** The advantages and drawbacks of epigenetic editing platforms.

**Function**	**Platform**	**Cas13**	**Advantages**	**Drawbacks**
A-to-I editing	REPAIR	dPspCas13b	Truncated REPAIR is suitable for AAV packaging	Low editing efficiency (<35%), low specificity
	xABE	dCas13x.1	Suitable for AAV packaging	Low editing efficiency (<30%)
C-to-U editing	RESCUE	dRanCas13b	Unclear	Low editing efficiency (<25%), low specificity
	xCBE	dCas13x.1	Suitable for AAV packaging	Modest editing efficiency (average 48%), low specificity
Methylation	TRM	dPspCas13b	Relative robust editing efficiency	dCas13-M3M14 exhibits low specificity, big size
Demethylation	dm^6^ACRISPR	dPspCas13b	Relative robust editing efficiency	Big size
A↔ m^6^A	PAMEC	dPguCas13b	Tunable through blue light	Modest editing efficiency (average 50%)
	PAMEC^R^	dPguCas13b	Tunable through blue light	Modest editing efficiency

### Limitations in the AAV Delivery System

AAV is characterized by low pathogenicity, high specificity, and high safety, as it does not integrate into the genome and various serotypes, making it the preferred delivery strategy for animal experiments and *in vivo* treatment (Flotte et al., 1992; Robbins and Ghivizzani, 1998). Nonetheless, the reshipment volume of adeno-associated viruses is limited to 4.7 kb (Grieger and Samulski, 2005; Figure 3). CRISPR-Cas is too big to be packaged into AAV, let alone for linking to effectors, which will narrow future clinical applications. To abate molecular weight, previous studies have truncated the C or N terminal of dCas13b-ADAR2 (E488Q/T375G) and found that △984–1090 could be packaged into AAV (Cox et al., 2017). With an increasing number of Cas13 orthologs being identified, sufficiently small and more effective effectors will be developed to bypass the package limit. Zhang’s lab members analyzed over 600 orthologs of Cas9 and demonstrated that the saCas9 system could be packaged into a single AAV vector and have comparable activity and off-target rates to those of the widely used spCas9 (Ran et al., 2015). Recently identified Cas13d and Cas13x are suitable for packaging into commonly used AAV system. Another strategy is to split dCas13 into different fragments according to their catalytic domains. Truong et al. (2015) developed a split-Cas9 system using a split-intein protein splicing system. The separated Cas9 fragments were fused with either an N-terminal intein fragment or a C-terminal intein fragment, which could be linked to each other, and self-catalytically integrated the two split Cas9 fragments into one Cas9 protein (Truong et al., 2015). A series of assays showed that the nuclease activity of this split-intein system was comparable to that of wild-type Cas9 and could be packaged, delivered, and function effectively. Inspired by the above system, Ma successfully resembled functional dCas9 fused with different transcription regulator domains for transcriptional control and developed logic AND circuits and sensory switches for the differential regulation of genes in response to cell type-specific miRNA (Ma et al., 2016). Base-editing platforms based on split-intein delivery system enable *in vivo* casual gene correction and markedly slow disease progression in various animal models (Villiger et al., 2018; Levy et al., 2020; Lim et al., 2020). Thus, previous studies regarding Cas9 optimization for AAV delivery can be used for reference.

## Conclusion and Future Perspective

The CRISPR-Cas13 system and its derivatives provide platforms for precisely altering RNA sequences and epi-transcriptomes in a temporary and controllable manner without permanently changing genomic sequences. It is extremely important to modulate RNA modifications, as these residues control RNA readouts, including RNA stability, translation efficiency, alternative splicing, and nuclear-cytoplasm translocation, all of which ultimately determine cellular physiological and pathological processes. Meanwhile, these platforms are also conducive to subtly study the epi-transcriptome because modifications in different transcripts have various effects; even modifications in the individual RNA vary in function. To date, the development of RNA engineering platforms is at the initial stages, and the majority are applied to the deamination and demethylation of m^6^A; although few studies have modulated m^6^Am, m^1^A and other RNA modifications are less abundant. In addition, some platforms are applied in disease models; RESCUE and REPAIR could correct many RNA sequence-related disorders and dm^6^ACRISPR can control disease processes via engineering modifications in oncogenes and tumor suppressor genes. Importantly, more attention should be paid to off-target possibilities, low editing efficiency and limitations in the AAV delivery system. Although there is much to be explored and addressed, we believe that both currently established and future CRISPR-based RNA editing systems have great potential to act as therapeutic strategies for various diseases.

## Author Contributions

TT wrote the manuscript. HH co-supervised the project. YH and YW contributed to editing the manuscript. PQ supervised the overall project and co-wrote the manuscript. All authors contributed to the article and approved the submitted version.

## Conflict of Interest

The authors declare that the research was conducted in the absence of any commercial or financial relationships that could be construed as a potential conflict of interest.

## Publisher’s Note

All claims expressed in this article are solely those of the authors and do not necessarily represent those of their affiliated organizations, or those of the publisher, the editors and the reviewers. Any product that may be evaluated in this article, or claim that may be made by its manufacturer, is not guaranteed or endorsed by the publisher.
